# Dynamic changes of exhaustion features in T cells during oral carcinogenesis

**DOI:** 10.1111/cpr.13207

**Published:** 2022-02-18

**Authors:** Wenqiang Xie, Jie Shen, Dikan Wang, Junyi Guo, Qunxing Li, Shuqiong Wen, Wenxiao Dai, Liling Wen, Huanzi Lu, Juan Fang, Zhi Wang

**Affiliations:** ^1^ Guanghua School of Stomatology Guangdong Provincial Key Laboratory of Stomatology Stomatological Hospital Sun Yat‐Sen University Guangzhou PR China; ^2^ School of Stomatology Zhejiang University School of Medicine Clinical Research Center for Oral Disease of Zhejiang Province Key Laboratory of Oral Biomedical Research of Zhejiang Province Cancer Center of Zhejiang University Hangzhou PR China; ^3^ Department of Stomatology Sun Yat‐sen Memorial Hospital Sun Yat‐sen University Guangzhou PR China

**Keywords:** cytokine secretion, inhibitory receptors, oral carcinogenesis, PD‐1 blockade, regulatory T cell (Treg), T cell exhaustion

## Abstract

**Objectives:**

This study aimed to clarify the dynamic changes of exhaustion features in T cells during oral carcinogenesis.

**Materials and Methods:**

Mice were randomly divided into 4NQO group and control group. The exhaustion features of CD4^+^ and CD8^+^ T cells of both groups were detected by flow cytometry. Furthermore, multiplex immunohistochemistry was used to evaluate the expression of inhibitory receptors in human normal, dysplastic, and carcinogenesis tissues. Finally, anti‐PD‐1 antibody treatment was performed at the early premalignant phase of oral carcinogenesis.

**Results:**

The proportion of naive T cells in 4NQO group was lower than those in control group, while the proportion of effector memory T cells was higher in 4NQO group. The expression of inhibitory receptors on CD4^+^ and CD8^+^ T cells increased gradually during carcinogenesis. In contrast, the secretion of cytokines by CD4^+^ and CD8^+^ T cells decreased gradually with the progression stage. Strikingly, those changes occurred before the onset of oral carcinogenesis. The expression of inhibitory receptors on T cells increased gradually as the human tissues progressed from normal, dysplasia to carcinoma. Interestingly, PD‐1 blockade at the early premalignant phase could reverse carcinogenesis progression by restoring T cell function.

**Conclusions:**

T‐cell dysfunction was established at the early premalignant phase of oral carcinogenesis; PD‐1 blockade at the early premalignant phase can effectively reverse T‐cell exhaustion features and then prevent carcinogenesis progression.

## Graphical abstract

T‐cell dysfunction was established at the early pre‐malignant phase of oral carcinogenesis. This process is accompanied by upregulation of multiple negative immunoregulatory receptor and T‐cell dysfunction.

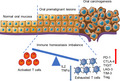

## 1 INTRODUCTION

Oral cancer, most commonly involves the tongue, is the sixth most frequent cancer in the world. In 2018, the total number of new cases of lip and oral cavity cancer worldwide was 354,864, showing an increased trend in morbidity compared with the relevant data from 2012.^1^, ^2^ Oral squamous cell carcinoma (OSCC) represents over 90% of all forms of malignancies in the oral cavity.^3^ Despite significant improvements made recently in the diagnosis and treatment of OSCC, the five‐year survival rate of OSCC still remains approximately 50%.^4^ Therefore, it is urgent to elucidate the underlying molecular and cellular mechanisms of oral carcinogenesis.

Most OSCC are thought to be derived from precancerous lesions through a multistage process including hyperplasia, dysplasia, carcinoma in situ, and invasive carcinoma, where molecular alterations accumulate over time and followed by observed epithelial changes. On the other hand, local immune cells are deeply involved in this multistage process, whether act as an effector arm against malignant transformation or contribute to disease progression. T cells, constituting an important element of both premalignant and tumor immune microenvironment, play a crucial role in preventing the development of OSCC. Retrospective studies have demonstrated that higher infiltration of T cells in oral premalignant lesions is associated with reduced susceptibility to OSCC.^5^, ^6^ Nonetheless, despite the presence of antigen‐specific T cells which are able to sense dysplastic epithelial cells in the oral premalignant lesions, the dysplastic cells cannot be eliminated.^7^ It has been proposed that the premalignant phase of oral carcinogenesis may equivalent to the equilibrium phase of cancer immunoediting and can be shifted toward OSCC as the adaptive immunity was impaired.^8^ However, whether and how the malignant transformation initiates and the dynamic changes in T‐cell states during oral carcinogenesis remain largely unknown.

Accumulating evidence indicates that T cells acquire an “exhaustion” state characterized by sustained expression of inhibitory receptors, progressive loss of effector functions, and distinct transcriptional and epigenetic profiles during the progression of cancer.^9^ Intriguingly, the dysfunctional tumor‐specific T cells found in the tumor microenvironment are already “imprinted” at the premalignant stage preceded tumor establishment due to continuous antigen encounter.^10^ It is reported that tumor‐specific neoantigens may arise at the premalignant phase and can be presented to the T cells.^11^ Similarly, in the context of oral carcinogenesis, oral premalignant lesions share several tumor antigens including EGFR, RAGE, and MUC1 with OSCC to stimulate T‐cell‐mediated immune response.^12^ Besides, PD‐L1, a coinhibitory receptor exerting its immunosuppressive effect via binding to PD‐1, was expressed on dysplastic epithelial cells and infiltrating cells in oral premalignant lesions and showed elevated expression in oral premalignant lesions that further progressed to cancer.^13^, ^14^ Therefore, we hypothesized that T‐cell dysfunction and immunosuppression occurred at the early premalignant phase of oral carcinogenesis. In addition, restoring T‐cell function at the early premalignant phase could prevent carcinogenesis progression.

In this study, we used a classical 4‐nitroquinoline‐1‐oxide (4NQO)‐induced murine OSCC model to mimic human oral cavity carcinogenesis. The oral epithelial lesions in mice were evaluated by hematoxylin and eosin (H&E) staining, and the dynamic changes of exhaustion features in T cells during the development of murine OSCC were monitored by flow cytometry. Our study has revealed that systemic T‐cell exhaustion emerges at the early stage of oral carcinogenesis and restoring T‐cell function at this stage could prevent oral carcinogenesis progression. This discovery may help in better understanding of the underlying mechanisms of OSCC pathogenesis and developing therapeutic strategies through remodeling T‐cell function at the early stage of oral carcinogenesis to prevent malignant transformation toward OSCC.

## 2 MATERIALS AND METHODS

### 2.1 Mice

Six‐week‐old female C57BL/6 mice were purchased from Beijing Vital River Laboratory Animal Technology Co., Ltd. All mice were maintained in a specific pathogen‐free environment. All animal study protocols were performed under the guidelines of the Institutional Animal Care and Use Committee of Sun Yat‐Sen University.

### 2.2 4NQO‐induced oral tumorigenesis

The 4NQO group was given sterile water containing 100 μg/ml 4‐Nitroquinoline‐1‐oxide (4NQO, Sigma‐Aldrich) for 16 weeks, while the control group was given sterile water to drink.^15^, ^16^ At week 16, the 4NQO water was changed into sterile water until the end of week 20. Mice were sacrificed at week 4, week 8, week 12, week 16, and week 20.

### 2.3 Antibody treatment

Anti‐mouse PD‐1 monoclonal antibody was a kind gift from Lieping Chen (Yale University School of Medicine). After 4NQO treatment for 12 weeks, the mice were randomly divided into anti‐PD‐1 group (αPD‐1 group) and control IgG group (Ctrl IgG group). The mice of αPD‐1 group were treated with 200 μg anti‐PD‐1 antibody intraperitoneally every week from 12 weeks to 16 weeks, while equivalent amounts of control IgG were administered intraperitoneally to the mice of Ctrl IgG group. All mice were sacrificed at week 20.

### 2.4 Histology and pathological analysis

Oral lesions were identified and photographed every 4 weeks. Next, oral lesions were harvested and fixed in 10% formalin for 24 h after euthanizing mice, then sectioned into 4‐μm slices. The slices were then stained with hematoxylin and eosin (H&E). After that, these slices were classified into multiple histopathological grades (normal, hyperplasia, dysplasia, and carcinoma) by 2 certified pathologists.

### 2.5 Flow cytometry

The Draining lymph node (Dln) and spleen were ground into a single cell suspension. The tongue tissue was cut into 1‐mm^3^ pieces and digested with DNase I (0.1 mg/ml) and collagenase IV (1 mg/ml) for 1 h. After red blood cells in the single cell suspension were lysed, single cell suspension was blocked by anti‐mouse FcR blocker for 30 min. The cells were further stained with antibodies in PBS with 2% FBS for 30 min at 4°C. Flow cytometry was performed according to a standardized protocol.^17^ Before intracellular cytokine staining, cells were stimulated in vitro with cell stimulation cocktail (TNB‐4975‐UL100, Tonbo Biosciences, USA) for 5 h at 37°C with 5% CO_2_. Antibodies for flow cytometry are listed in Table S1. Flow cytometric analyses were performed using BD LSRFFortessa (BD Biosciences), and data were analyzed using FlowJo 10.5.3 software. The fluorescence minus one (FMO) was performed as the flow cytometric controls for establishing gates. The DownSample plugin of FlowJo 10.5.3 software was used to extract 3000 events in CD3^+^ subgroup of each sample.^18^ After concatenating the extracted data of each group, t‐SNE plugin was used to obtain t‐SNE graph.

### 2.6 Multiplex immunohistochemistry

For multiplex immunohistochemistry (mIHC) staining, tissues from 5 OSCC samples, 5 oral leukoplakia (OLK) samples and 5 adjacent normal tissues samples were obtained during surgical resection at the Hospital of Stomatology, Sun Yat‐sen University (Guangzhou, Guangdong, P.R. China), and written informed consent was obtained from each patient. The mIHC staining was performed with the Opal fluorescent IHC Kit (NEL811001KT, PerkinElmer).^19^ At first, antigen retrieval was performed under high temperature and high pressure for 10 min with Tris‐EDTA (pH = 9.0). Subsequently, the slices were incubated with primary antibody for 16 h (4°C). The primary antibodies were listed as follows: anti‐human CD4 (EP204, CST), anti‐human CD8 (D8A8Y, CST), anti‐human PD‐1 (D4W2J, CST), anti‐human TIM‐3 (D5D5R, CST), and anti‐human LAG‐3 (D2G40, CST). Then the slices were incubated with secondary‐HRP and Opal TSA dyes for 10 min (RT) and 20 min (RT), respectively. Afterward, the other cycles of staining were implemented. The Opal detection fluorophores were as follows: CD4‐Opal 650, CD8‐Opal 620, PD‐1‐Opal 570, TIM‐3‐Opal 540, and LAG‐3‐Opal 520. DAPI was applied for nuclear staining. The immunofluorescence images were captured and analyzed with Vectra 3.0 imaging system and inForm image analysis software (Vectra 3.0; Perkin Elmer).

### 2.7 Statistical analysis

All statistical analyses were implemented with GraphPad Prism 7.0. Fisher's exact test was used to compare the proportion of oral lesions of Ctrl IgG group and αPD‐1 group. Differences in flow cytometric results were evaluated with Student's *t* test. The results of mIHC were evaluated with one‐way analysis of variance (ANOVA). All analyses were two‐tailed, and *p* < 0.05 was considered significant.

## 3 RESULTS

### 3.1 4NQO induced the development of OSCC in mice

**FIGURE 1:**
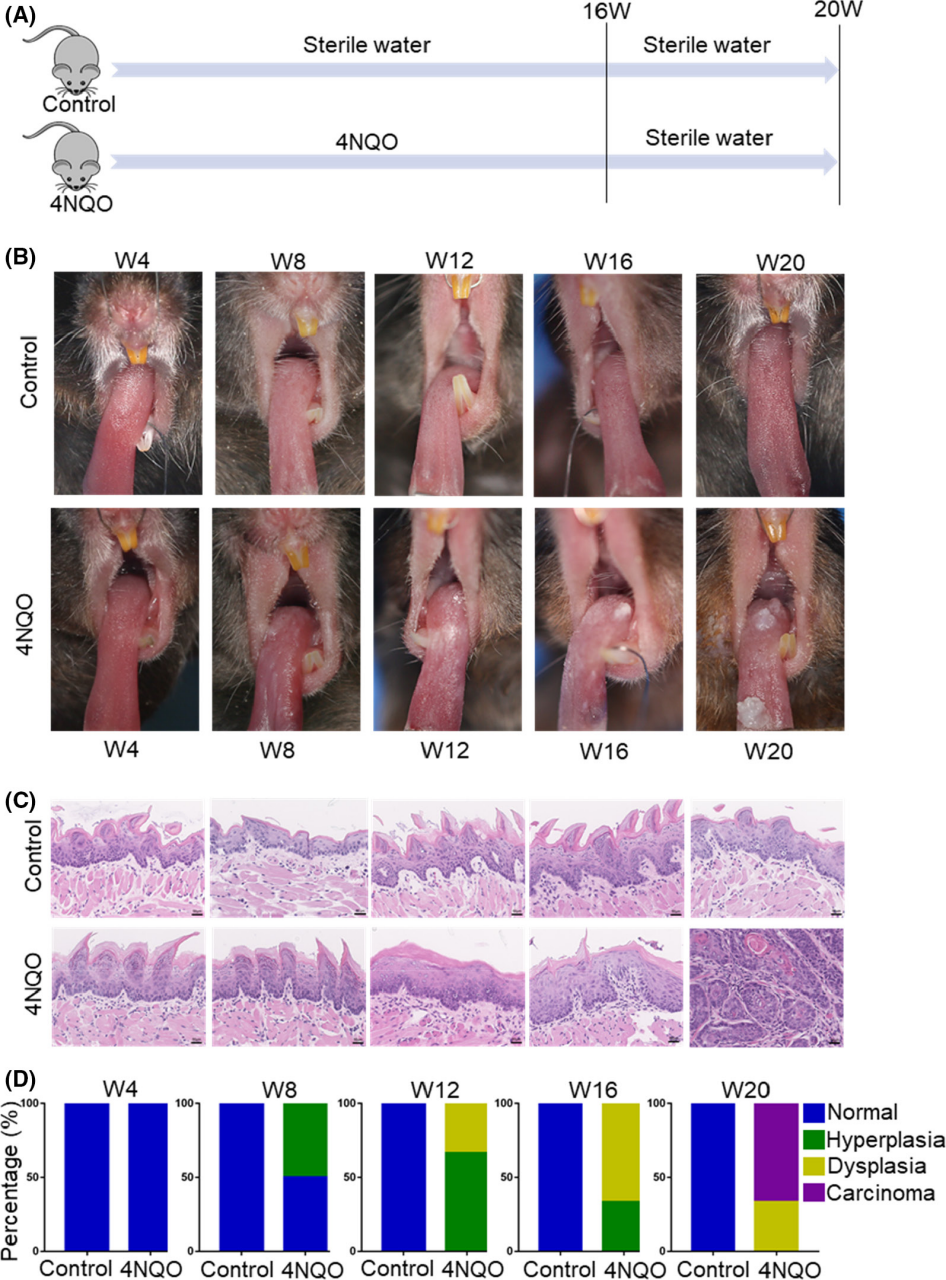
4NQO administration resulted in precancerous and cancerous lesions in mice. (A) Mice were fed with 4NQO or sterile water for 16 weeks and then given sterile water until week 20. (B) Representative pictures of precancerous and cancerous lesion formation every 4 weeks in 4NQO group and control group. (C) Representative hematoxylin and eosin (H&E) of 4NQO group and control group. (D) Changes of the proportion of oral lesions in 4NQO group. W4, week 4; W8, week 8; W12, week 12; W16, week 16; W20, week 20

To investigate T‐cell exhaustion during the process of OSCC, we induced precancerous and cancerous lesions in mice with 4NQO (Figure 1A). Six mice in the 4NQO group and the control group were sacrificed every 4 weeks during the induction process. Then, the oral mucosal lesions of mice were detected. With the increase in time of 4NQO induction, hyperplasia, dysplasia, and carcinoma in the oral mucosa of mice were observed at week 8, week 12, and week 20, respectively (Figure 1B–D). Therefore, in this study, the oral mucosa of mice at week 4, week8, and week20 was in normal, hyperplasia, and carcinoma stage, respectively, while the oral mucosa of mice at week 12 and week16 was in dysplasia stage.

### 3.2 The differentiation states of T cells changed during the development of OSCC

**FIGURE 2:**
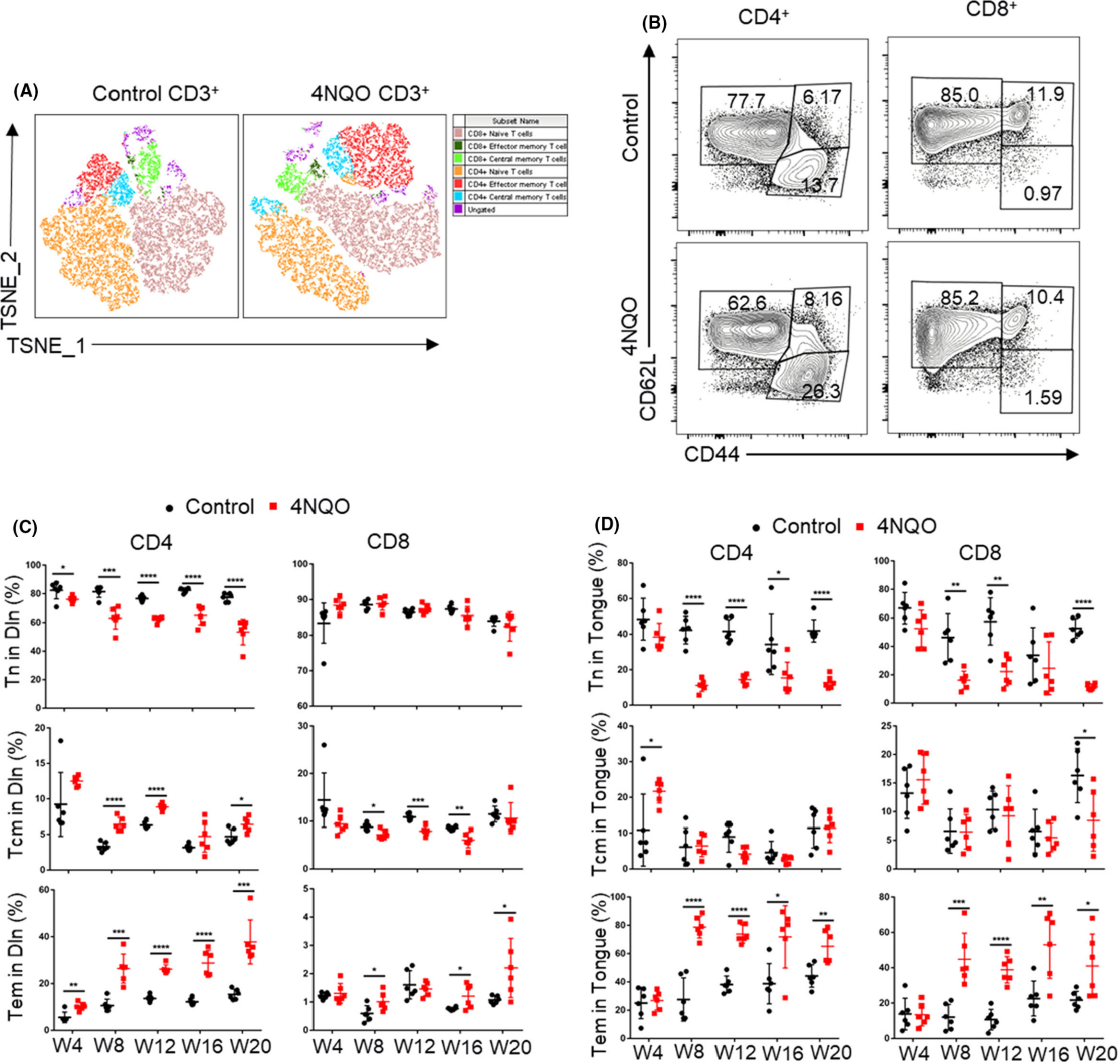
CD4^+^ and CD8^+^ T‐cell differentiation states assessed by CD44 and CD62L expression. (A) T‐Distributed Stochastic Neighbor Embedding (T‐SNE) maps of T cells differentiation states in control and 4NQO mice. The parameters including CD4, CD8, CD44, and CD62L were incorporated in the analysis. (B) Representative flow cytometry of CD4^+^ and CD8^+^ T‐cell differentiation states assessed by CD44 and CD62L expression. (C and D) Statistical results of CD4^+^ and CD8^+^ T‐cell differentiation states in draining lymph node (C), and tongue (D) of mice. Tn, naïve T cells; Tcm, central memory T cells; Tem, effector memory T cells; Dln, draining lymph node; W4, week 4; W8, week 8; W12, week 12; W16, week 16; W20, week 20. *p < 0.05, **p < 0.01, ***p < 0.001, ****p < 0.0001

The differentiation states of CD4^+^ and CD8^+^ T cells in the spleen, draining lymph node (Dln), and tongue were detected with flow cytometry every 4 weeks (Figure S1). The proportion of effector memory T cells (Tem, CD44^+^ CD62L^−^) and central memory T cells (Tcm, CD44^+^ CD62L^+^) was higher in 4NQO group than in control group, while the proportion of naive T cells (Tn, CD44^−^ CD62L^+^) was lower in 4NQO group than in control group (Figure 2A).

Compared with control group, 4NQO group showed a higher proportion of CD4^+^ Tem in Dln at week 4 and in tongue and spleen at week 8. 4NQO group showed a higher proportion of CD4^+^ Tcm in Dln and spleen at week 8 and showed a higher proportion in tongue at week 4. Conversely, 4NQO group showed a lower proportion of CD4^+^ Tn in Dln, tongue, and spleen at the week 4, week 8, and week 12, respectively (Figure 2B–D and Figure S2A,B).

Similarly, the percentage of CD8^+^ Tem in tongue and Dln was significantly higher in 4NQO group compared with control group at week 8, but there was no significant difference in spleen. 4NQO group showed a lower proportion of CD8^+^ Tn in tongue at week 8, but there was no significant difference in Dln and spleen. Compared with the control group, 4NQO group showed a lower proportion of CD8^+^ Tcm in Dln and tongue at week 8 and week 20, respectively, but there was no significant difference in spleen (Figure 2B–D and Figure S2A,B).

### 3.3 The expression of inhibitory receptors on T cells gradually increased during the process of OSCC development

**FIGURE 3:**
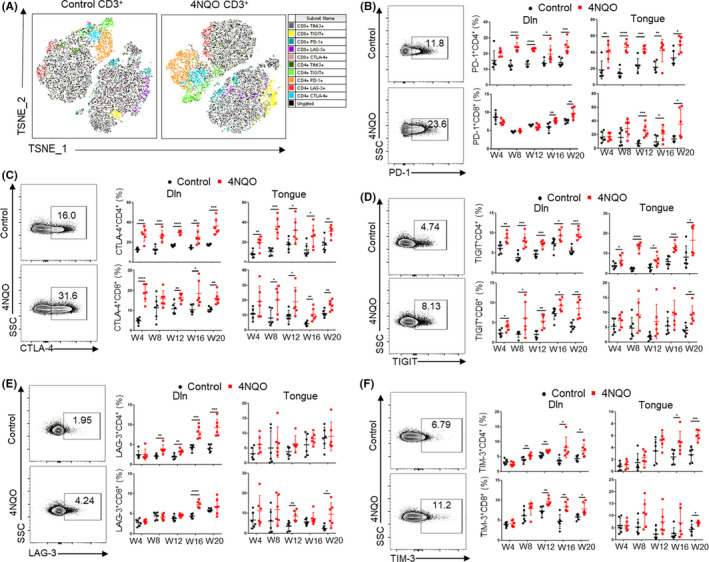
Expression of inhibitory receptors on T cells increased gradually with the development of OSCC. (A) T‐SNE plots of inhibitory receptors expression on T cells in control and 4NQO mice. The parameters including CD4, CD8, PD‐1, CTLA‐4, TIGIT, LAG‐3, and TIM‐3 were incorporated in the analysis. (B, C, D, E, and F) Expression of PD‐1 (B), CTLA‐4 (C), TIGIT (D), LAG‐3 (E), and TIM‐3 (F) on T cells in Dln and tongue during the development of OSCC. W4, week 4; W8, week 8; W12, week 12; W16, week 16; W20, week 20. *p < 0.05, **p < 0.01, ***p < 0.001, ****p < 0.0001

The expression of inhibitory receptors such as PD‐1, CTLA‐4, TIGIT, LAG‐3, and TIM‐3 increased in the process of T‐cell exhaustion. Flow cytometry was used to detect the expression of inhibitory receptors in spleen, Dln, and tongue at different stages during OSCC progression. The results showed that the expressions of PD‐1, CTLA‐4, and TIGIT on CD4^+^ T cells of Dln and tongue in 4NQO group compared with control group were significantly up‐regulated at week 4 and week 8, respectively (Figure 2A–D). The expression of CTLA‐4 in spleen CD4^+^ T cells of 4NQO group was significantly up‐regulated than that of control group at week 4, while the expression of PD‐1 and TIGIT in spleen CD4^+^ T cells were not significantly different between two groups (Figure S3A–C). The expression of LAG‐3 and TIM‐3 in CD4^+^ T cells of 4NQO group was significantly up‐regulated at week 8 in Dln compared with that of control group. TIM‐3 expression on CD4^+^ T cells in tongue began to show statistical difference at the week16, but LAG‐3 expression on CD4^+^ T cells in tongue had no significant difference between these two groups at all time points (Figure 3E,F). At the same time, LAG‐3 and TIM‐3 expressions in spleen on CD4^+^ T cells showed difference at week 12 (Figure S3D,E).

Compared with control group, 4NQO group showed significantly up‐regulated expression of PD‐1 on CD8^+^ T cells at week 16 in Dln and at week 12 in tongue, but there was no significant difference in spleen (Figure 3B and Figure S3A). The expression of CTLA‐4 and TIGIT on CD8^+^ T cells in Dln of 4NQO group was significantly up‐regulated at week 4 compared with that of control group. CTLA‐4 and TIGIT expression on CD8^+^ T cells in the tongue of 4NQO group was up‐regulated at week 8 and week 20, respectively (Figure 3C,D). The expression of CTLA‐4 and TIGIT on CD8^+^ T cells in spleen was significantly different at week 20 and week 12, respectively (Figure S3B,C). The expression of LAG‐3 and TIM‐3 on CD8^+^ T cells in Dln of 4NQO group was significantly up‐regulated at week 16 and week 12, respectively, compared with that of control group. Besides, there was a significant difference in LAG‐3 and TIM‐3 expression on CD8^+^ T cells in tongue between two groups at week 12 and week 20 (Figure 3E,F), but there was no significant difference in LAG‐3 and TIM‐3 expression on CD8^+^ T cells in spleen (Figure S3D,E).

From the above results, it can be found that the up‐regulation of inhibitory receptors on CD4^+^ T cells appears earlier and more obvious than that on CD8^+^ T cells, indicating that CD4^+^ T cells are more vulnerable to develop an exhaustion phenotype during the development of OSCC than CD8^+^ T cells.

### 3.4 The cytokine secretion of T cells decreased gradually during the development of OSCC

**FIGURE 4:**
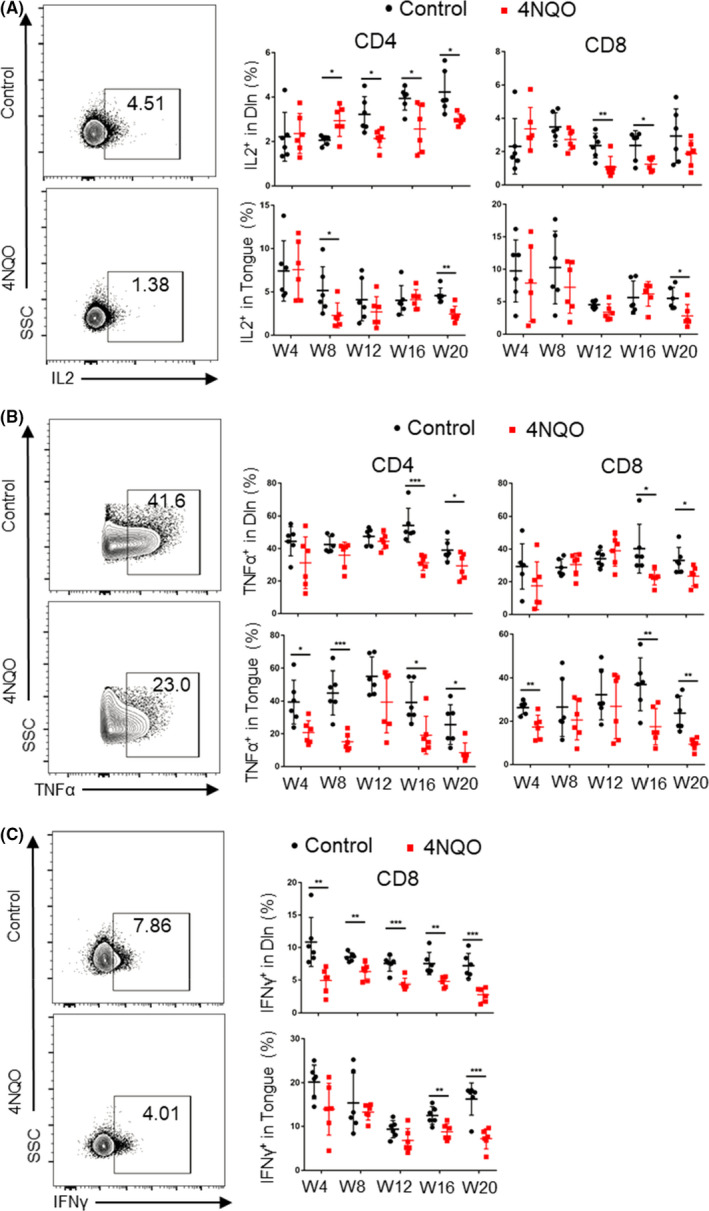
Cytokine secretion on T cells decreased gradually during the development of OSCC. Percentages of T cells secreting IL2 (A), TNFα (B), and IFNγ (C) in Dln and tongue during the development of oral carcinogenesis. W4, week 4; W8, week 8; W12, week 12; W16, week 16; W20, week 20

The exhaustion of T cells is also characterized by the progressive reduction in cytokine secretion. Our results showed that the secretion of IL‐2 by CD4^+^ T cells in Dln and tongue of 4NQO group was significantly lower than that of control group from week 8 (Figure 4A), but there was no significant difference in spleen (Figure S4A). The production of TNFα by CD4^+^ T cells in Dln and tongue of 4NQO group was significantly lower than that of control group at week 16 and week 4, respectively (Figure 4B), while there was no significant difference about the secretion of TNFα on CD4^+^ T cells in spleen (Figure S4B).

In CD8^+^ T cells, the secretion of IL2 in Dln and tongue of 4NQO group was significantly lower than that of control group at week 12 and week 20, respectively (Figure 4A), while there was no significant difference in spleen (Figure S4A). The secretion of TNFα in Dln, tongue, and spleen of 4NQO group was significantly lower than that of control group from week 16, week 4, and week 12, respectively (Figure 4B and Figure S4B). The secretion of IFNγ in Dln, tongue, and spleen of 4NQO group was significantly lower than that of control group at week 4, week 16, and week 12, respectively (Figure 4C and Figure S4C).

### 3.5 The proportion of CD4^+^ T‐cell subsets changed and MDSCs were enriched during oral carcinogenesis

**FIGURE 5:**
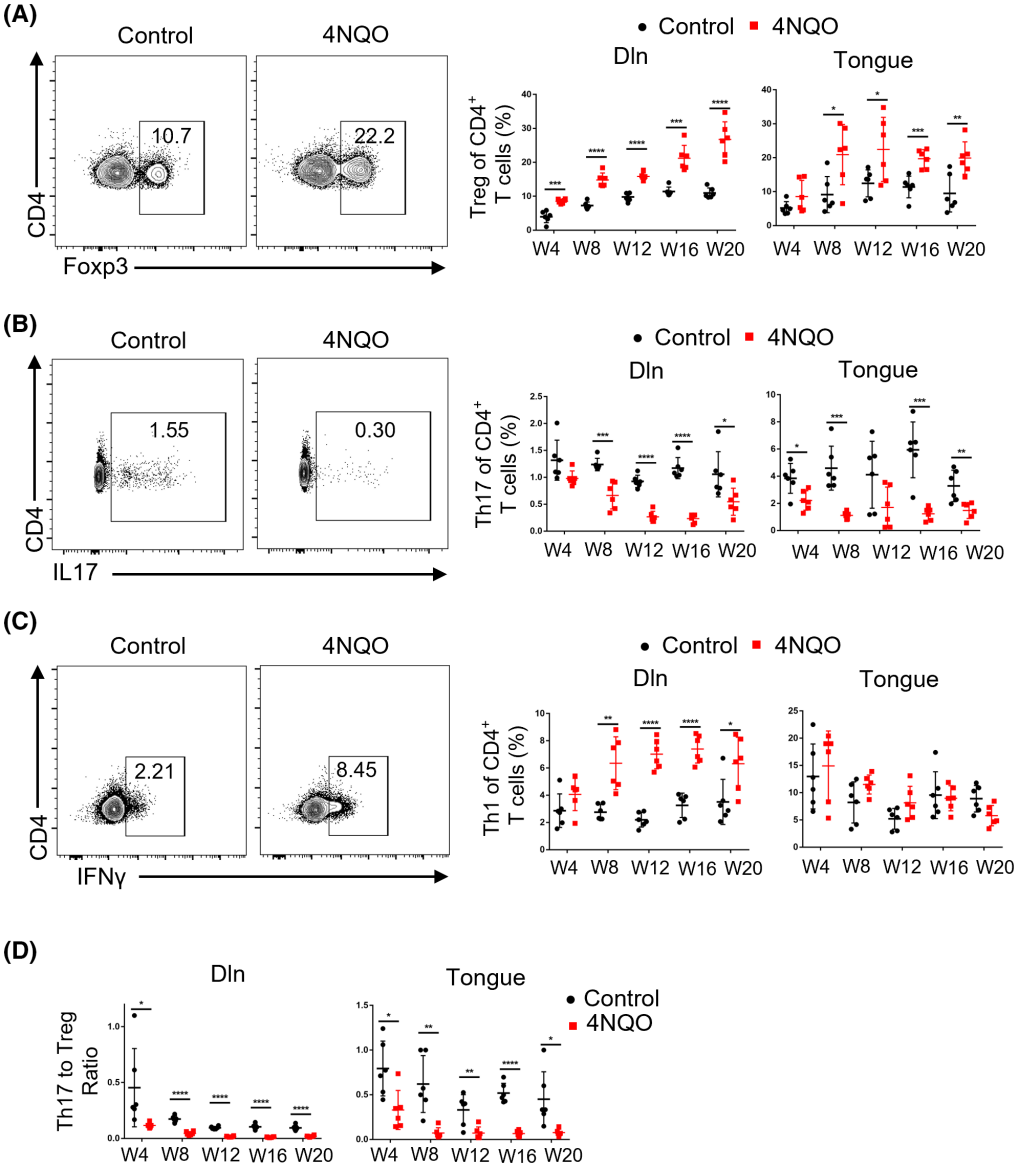
FIGURE Proportion of Treg and Th1 increased, while the proportion of Th17 decreased during the development of OSCC. (A–C) Representative flow cytometric analysis and the percentages of Treg (A), Th17 (B), and Th1 (C) in Dln and tongue were shown. (D) Representative the ratio of Th17 to Treg were shown. W4, week 4; W8, week 8; W12, week 12; W16, week 16; W20, week 20. *p < 0.05, **p < 0.01, ***p < 0.001, ****p < 0.0001

It is known that CD4^+^ T cells comprise various subtypes, including Th1, Th2, Th17, and Treg, etc. Among them, Th1, Th17, and Treg are the most important cell subsets in carcinogenesis. Therefore, we detected the proportion of Th1, Treg, and Th17 in spleen, Dln, and tongue with flow cytometry. The results showed that the proportion of Treg in spleen, Dln, and tongue of 4NQO group was higher than that of control group at the early stage of induction, and the difference was more obvious with the extension of induction (Figure 5A and Figure S5A). The proportion of Th17 in spleen, Dln, and tongue of 4NQO group was lower than that of control group (Figure 5B and Figure S5B). The proportion of Th1 in spleen and Dln of 4NQO group was higher than that of control group, but no significant difference was observed between two groups in tongue (Figure 5C and Figure S5C). The ratio of Th17 to Treg of 4NQO group decreased from week 4 in spleen, Dln and tongue (Figure 5D and Figure S5D).

MDSCs are also important subsets of immunosuppressive cells during tumor development. However, since the number of T cells isolated from lingual lesions is too small to separate parts for MDSCs detection, we only detected MDSCs in spleen and Dln. The results showed that the proportion of MDSCs of 4NQO group (especially in spleen) was higher than that of control group (Figure S6).

### 3.6 T‐cell exhaustion was also associated with the development of human OSCC

**FIGURE 6:**
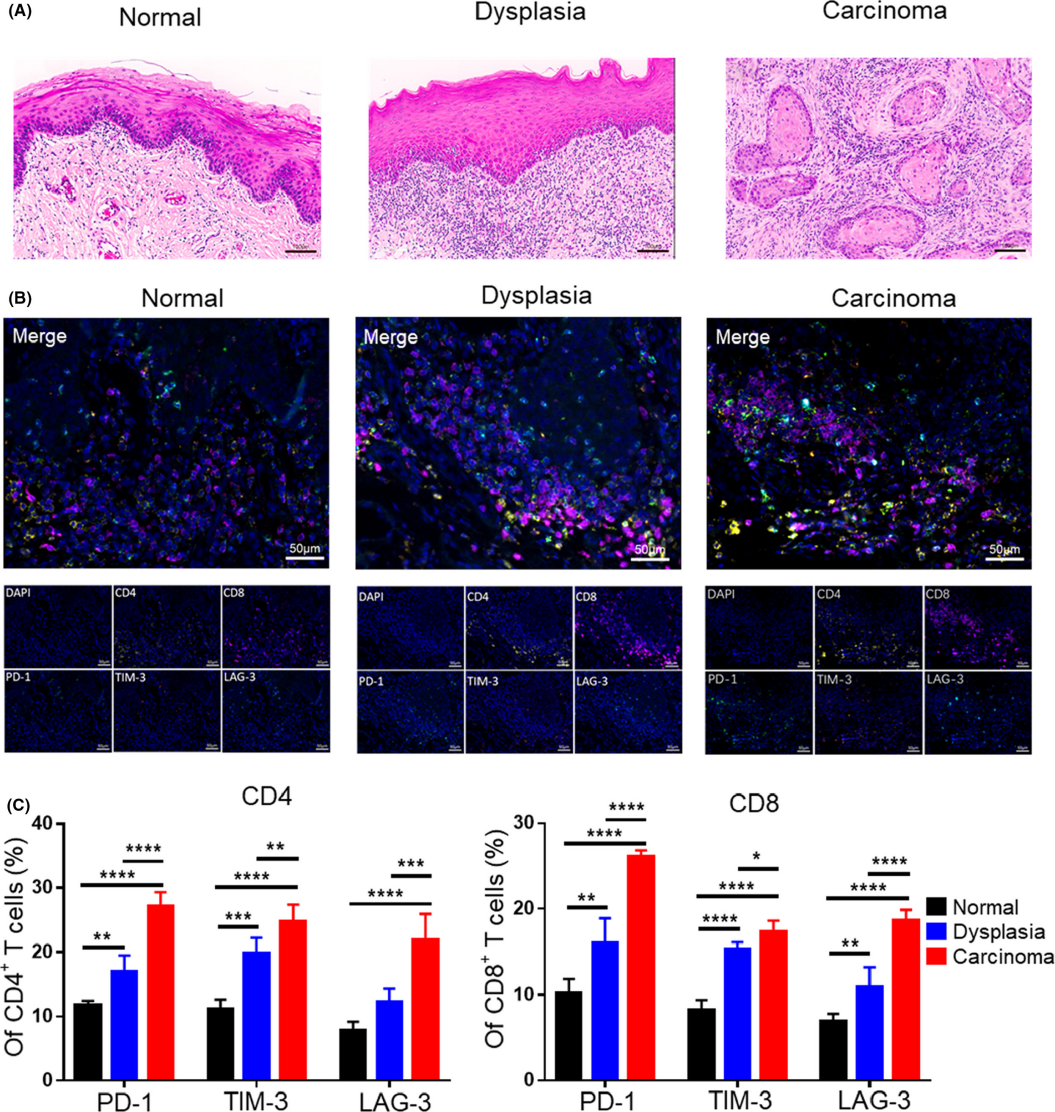
Expression of inhibitory receptors on T cells increased during the development of human OSCC. (A) Representative hematoxylin and eosin (H&E) staining images of human normal, dysplasia, and carcinoma tissues. (B) Representative multiplex immunohistochemistry (mIHC) images showing expression of inhibitory receptors on T cells of normal, dysplasia, and carcinoma tissues by simultaneous staining of CD4^+^ T cells (CD4, yellow), CD8^+^ T cells (CD8, purple), PD‐1 (PD‐1, green), TIM‐3 (TIM‐3, red), LAG‐3 (LAG‐3, cyan), and the nuclear stain DAPI (blue). (C) Statistical graph showing the expression of inhibitory receptors on CD4^+^ or CD8^+^ T cells in mIHC. *, *p* < 0.05; **, *p* < 0.01; ***, *p* < 0.001; and ****, *p* < 0.0001

To further validate the changes in exhausted features of T cells during the development of OSCC, we performed multiple immunohistochemistry (mIHC) on human normal, dysplasia, and carcinoma tissues. We found that the expression of inhibitory receptors such as PD‐1, TIM‐3, and LAG‐3 increased successively from normal, dysplasia to carcinoma tissues (Figure 6A–C). This phenomenon was consistent with observations in 4NQO‐induced mouse carcinogenesis model.

### 3.7 PD‐1 blockade at the early premalignant phase can effectively prevent oral malignant lesions progression by restoring T‐cell function

**FIGURE 7:**
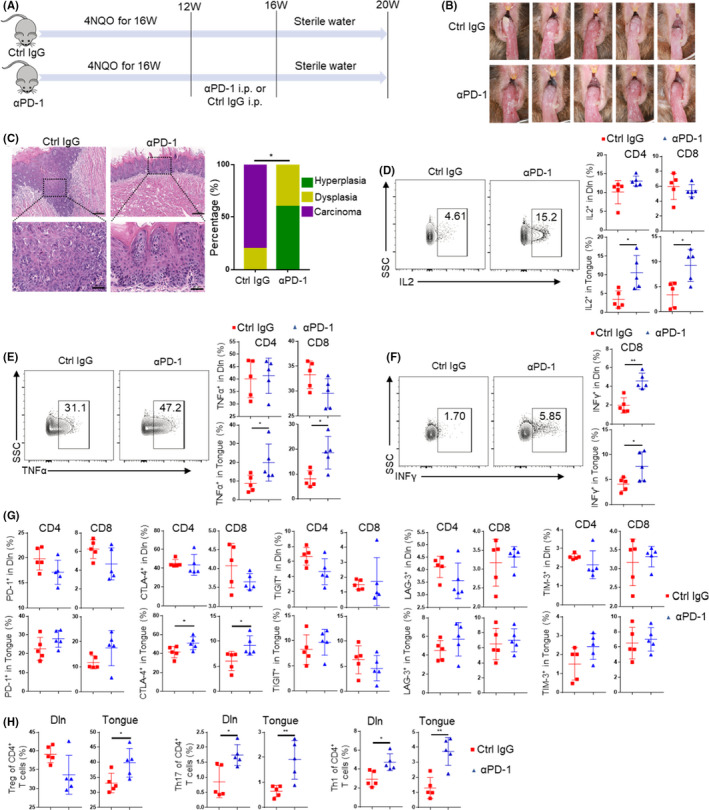
PD‐1 blockade prevents oral malignant lesions progression by reversing T cell exhaustion features. (A) Mice were fed with 4NQO for 16 weeks and then given sterile water until week 20. Anti‐PD‐1 antibody (αPD‐1) or control IgG (Ctrl IgG) treatment was performed from week 12 to week 16. (B) Photograph of lingual mucosal lesion of Ctrl IgG group and αPD‐1 group. (C) Representative H&E and proportion of oral lesions of Ctrl IgG group and αPD‐1 group (upper scale bars:200 μm; lower scale bars:50 μm). (D‐F) Representative flow cytometric analysis and percentages of T cells secreting IL2 (D), TNFα (E) and IFNγ (F) in Dln and tongue of Ctrl IgG group and αPD‐1 group. (G) Expression of PD‐1, CTLA‐4, TIGIT, LAG‐3, and TIM‐3 on T cells in Dln and tongue of Ctrl IgG group and αPD‐1 group. (H) The percentages of Treg, Th17 and Th1 in Dln and tongue of Ctrl IgG group and αPD‐1 group. *p < 0.05, **p < 0.01

Since T‐cell dysfunction was established at the early premalignant phase of oral carcinogenesis, we continued to explore whether PD‐1 blockade at the early premalignant phase could prevent oral carcinogenesis progression. Anti‐PD‐1 antibody (αPD‐1) or control IgG (Ctrl IgG) treatment was performed from week 12 to week 16 in 4NQO‐induced OSCC models (Figure 7A). From photos and H&E staining of oral lesions, it could be found that PD‐1 blockade at the early premalignant phase can effectively prevent oral malignant lesions progression (Figure 7B,C). Among them, most of the oral lesions of Ctrl IgG group progressed to carcinoma, while most of the oral lesions of αPD‐1 group stayed at hyperplasia stage (Figure 7C).

Flow cytometric analysis showed that the secretion of IL2 and TNFα by CD4^+^ or CD8^+^ T cells in tongue of αPD‐1 group was significantly higher than that of Ctrl IgG group (Figure 7D,E). However, there was no significant difference about the secretion of IL2 and TNFα by CD4^+^ or CD8^+^ T cells in Dln or spleen (Figure 7D,E and Figure S7A). Furthermore, the secretion of IFNγ by CD8^+^ T cells in Dln and tongue of αPD‐1 group was significantly higher than that of Ctrl IgG group, while there was no difference in spleen (Figure 7F and Figure S7A). The expression of CTLA‐4 on CD4^+^ or CD8^+^ T cells in tongue up‐regulated after αPD‐1 treatment, while the expression of other inhibitory receptors on CD4^+^ or CD8^+^ T cells did not significantly change after αPD‐1 treatment (Figure 7G and Figure S7B). Then we found that the proportion of Treg in tongue increased significantly after αPD‐1 treatment. The proportion of Th17 and Th1 in Dln and tongue also increased significantly after αPD‐1 treatment. However, T‐cell subsets except Th17 did not change significantly in the spleen αPD‐1 treatment (Figure 7H and Figure S7C). No significant changes were found in differentiation states of CD4^+^ or CD8^+^ T cells in Dln, tongue, and spleen between αPD‐1 group and Ctrl IgG group (Figure S7D).

## 4 DISCUSSION

Although many studies have investigated the role of T‐cell‐mediated immune response in OSCC pathogenesis,^17^, ^20^, ^21^ T‐cell states in the oral premalignant lesions and its dynamic changes during oral carcinogenesis are poorly understood. In this study, we aimed to clarify the dynamic changes of exhaustion features in T cells as well as other involved immune events during oral carcinogenesis. We found that T cells became dysfunctional at the early stage of epithelial dysplasia. We also found an increase in Treg and Th1 proportion and a decrease in Th17 proportion as disease progresses. Interestingly, anti‐PD‐1 antibody treatment at the early premalignant phase could effectively prevent the development of oral carcinogenesis by restoring T‐cell function.

4NQO‐induced OSCC murine model, which can simulate the dynamic process of carcinogenesis from normal to hyperplasia, then to dysplasia, and finally to carcinoma, is currently recognized as the most suitable model to investigate human OSCC multi‐step carcinogenesis.^22^, ^23^ Although the tumor formation time of 4NQO‐induced OSCC model is much longer than a transplanting neoplasm model, the whole carcinogenesis process is well defined and easy to be observed. Therefore, it is an excellent model for studying the initiation and progression of T‐cell exhaustion and the interaction between epithelial cells and local immune cells in the context of oral carcinogenesis.

T‐cell exhaustion is a states of T cell dysfunction. We demonstrated that CD4^+^ and CD8^+^ T cells acquire their exhausted features at the early stage of oral carcinogenesis prior to tumor establishment, which could in turn accelerate the development of oral carcinogenesis. Furthermore, PD‐1 blockade at the early stage of oral carcinogenesis could effectively prevent oral malignant progression. We and other researchers previously reported that although PD‐1 blockade can effectively prevent the occurrence of OSCC, the phenomenon of resistance against anti‐PD‐1 therapy still exists and therefore some mice still progress to OSCC.^17^, ^20^, ^24^, ^25^, ^26^, ^27^ In this study, most of the oral lesions of αPD‐1 group stayed at hyperplasia stage, and no oral lesions in αPD‐1 group progressed to OSCC. The reason for this difference might be that the dysfunctional T cells are still in “pre‐exhausted” states. These “pre‐exhausted” T cells were more sensitive to PD‐1 blockade therapy than terminal exhausted T cells.^28^ These results provided a reference for checkpoint inhibitors blockade in treatment of oral premalignant lesions. An ongoing phase II trial (NCT04504552) aimed to treat high‐risk oral premalignant lesions with checkpoint inhibitors.

Moreover, previous studies have revealed that the anti‐tumor function of CD4^+^ T cells is not weaker than that of CD8^+^ T cells.^29^ Here, we found that even in the premalignant lesions, CD4^+^ T cells were more likely to be activated by antigen stimulation than CD8^+^ T cells demonstrated by an increased proportion of effector T cells and Th1 cells (Figures 2 and 5). However, CD4^+^ T cells also showed up‐regulated inhibitory receptors earlier than CD8^+^ T cells. These results indicated that reversal of CD4^+^ T cells exhaustion at the premalignant stage was also expected to be an effective strategy for OSCC prevention.

Tregs infiltration within premalignant microenvironment was significantly associated with the increased risk of cancerization to OSCC.^30^ The Th17/Treg ratio was reported to be a potential diagnostic indicator for malignant transformation to OSCC.^31^ Besides, promoting Th17 phenotype and inhibiting its dynamic shift to Treg phenotype in premalignant oral lesions might slow disease progression.^32^, ^33^ However, 4NQO group showed significantly lower Th17/Treg ratio compared with control group. MDSCs were also found to be enriched in 4NQO group. It was reported that MDSCs were capable of inducing Treg,^34^ which might result in a positive feedback loop that augmented immunosuppression. These findings were consistent with a recent study.^35^ In addition, we found that the expression of CTLA‐4 on T cells and the proportion of Treg increased significantly after anti‐PD‐1 therapy, which may offset the effect of therapy. These results suggested that PD‐1 blockade combined with CTLA‐4 blockade and Treg clearance may display a better therapeutic effect.

## 5 CONCLUSIONS

Our study characterized the dynamic changes in T cell exhausted features during OSCC development, which provided direct evidence that T cell exhaustion occurred at the early stage of oral carcinogenesis. Interestingly, anti‐PD‐1 antibody treatment at the early premalignant phase could more effectively prevent the development of oral carcinogenesis than treatment after tumor formation.

## CONFLICT OF INTEREST

The authors have declared that no conflict of interest.

## AUTHOR CONTRIBUTIONS

Zhi Wang conceptualized the study. Wenqiang Xie, Jie Shen, Dikan Wang, and Qunxing Li acquired most data. Junyi Guo, Jie Shen, and Shuqiong Wen contributed to revision of the manuscript. Wenxiao Dai, Liling Wen, Huanzi Lu and Fang Juan involved in investigation. Zhi Wang and Fang Juan involved in funding acquisition.

## Supporting information

Supplementary materials

## Data Availability

The authors declare that all data supporting the findings of this study are available on reasonable request from the corresponding author.
