# Supplementary material for: Dynamic changes of exhaustion features in T cells during oral carcinogenesis
Source: Cell Prolif. 2022 Feb 18;55(4):e13207. doi: 10.1111/cpr.13207 (PMC9055910; doi:10.1111/cpr.13207)
Supplement: Supplementary file 1 — Supplementary materials [file CPR-55-e13207-s001.docx]

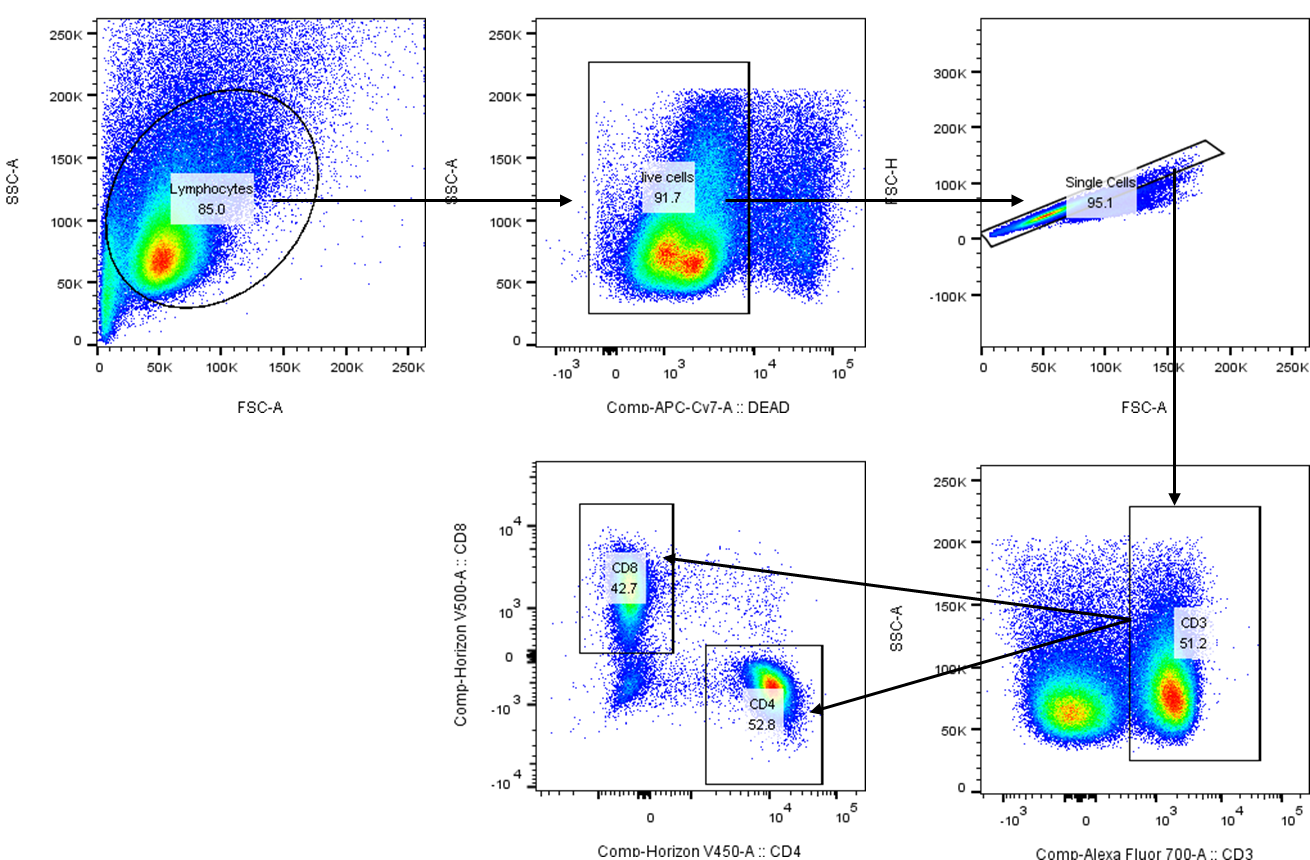


**Figure S1. Flow cytometry gating strategy.**


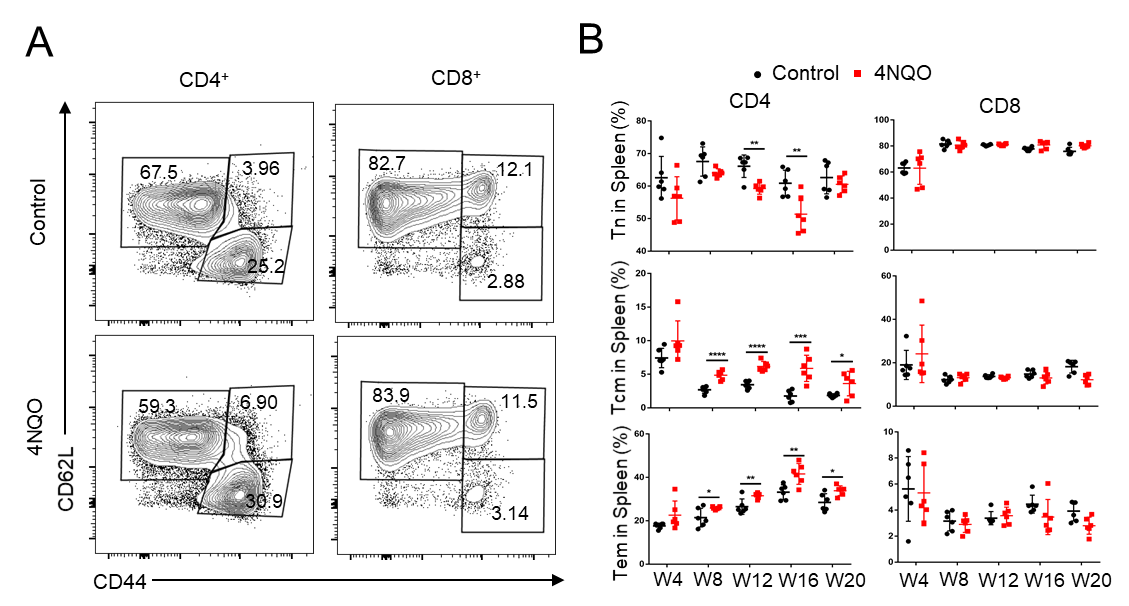


**Figure S2.** **Differentiation states of CD4^+^ and CD8^+^ T cells in spleen.** (A) Representative flow cytometry of CD4^+^ and CD8^+^ T cells differentiation states assessed by CD44 and CD62L expression. (B) Statistical results of CD4^+^ and CD8^+^ T cell differentiation states in spleen. W4, week 4; W8, week 8; W12, week 12; W16, week 16; W20, week 20. **p*<0.05, ***p*<0.01, ****p*<0.001, *****p*<0.0001.


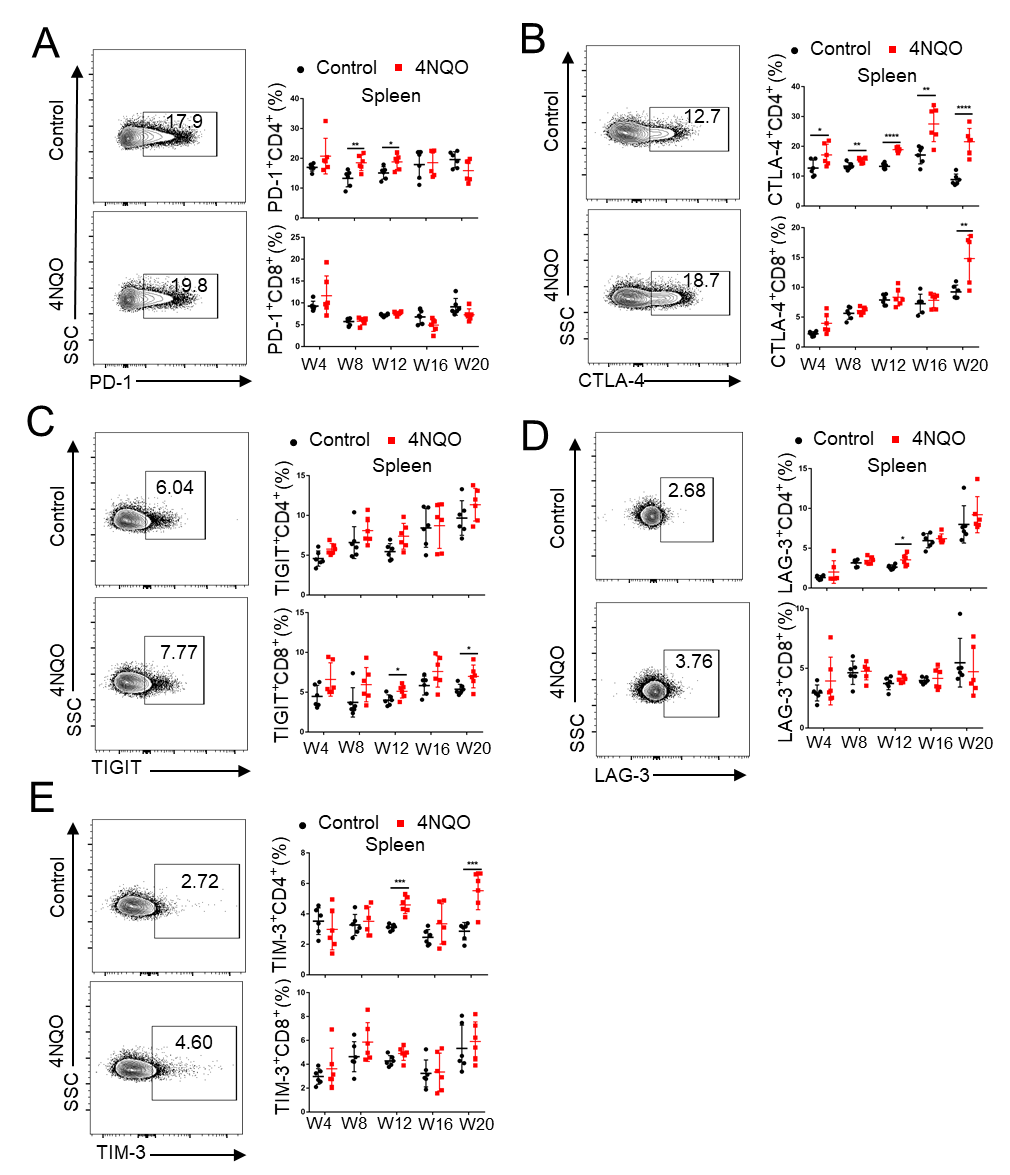


**Figure S3. The expression changes of inhibitory receptors on T cells in spleen during the development of OSCC.** (A-E) Expression of PD-1 (A), CTLA-4 (B), TIGIT (C), LAG-3 (D), and TIM-3 (E) on T cells in spleen during the development of OSCC. W4, week 4; W8, week 8; W12, week 12; W16, week 16; W20, week 20. **p*<0.05, ***p*<0.01, ****p*<0.001, *****p*<0.0001.

**
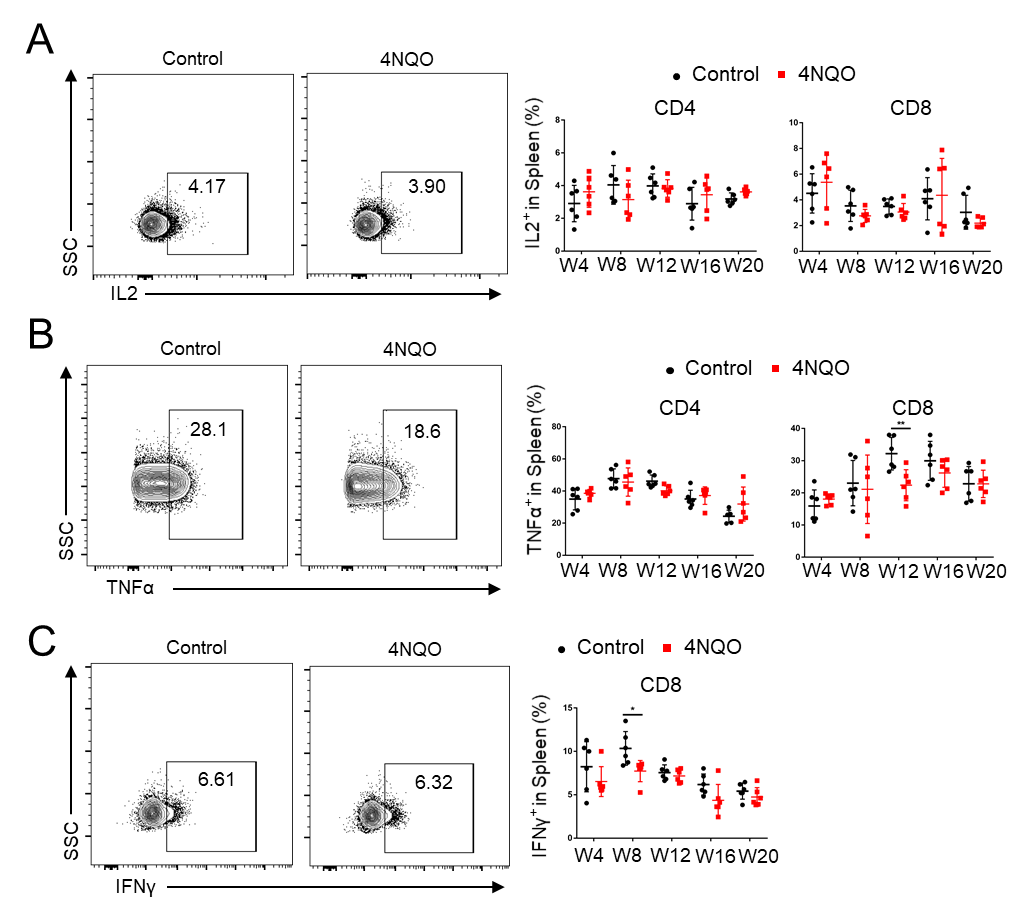
**

**Figure S4. The changes of cytokine secretion on T cells in spleen during the development of OSCC.** (A-C) Percentages of CD4^+^ and CD8^+^ T cells secreting IL2 (A), TNFα(B) and IFNγ (C) in spleen during the development of oral cancer. W4, week 4; W8, week 8; W12, week 12; W16, week 16; W20, week 20. **p*<0.05, ***p*<0.01, ****p*<0.001, *****p*<0.0001.

**
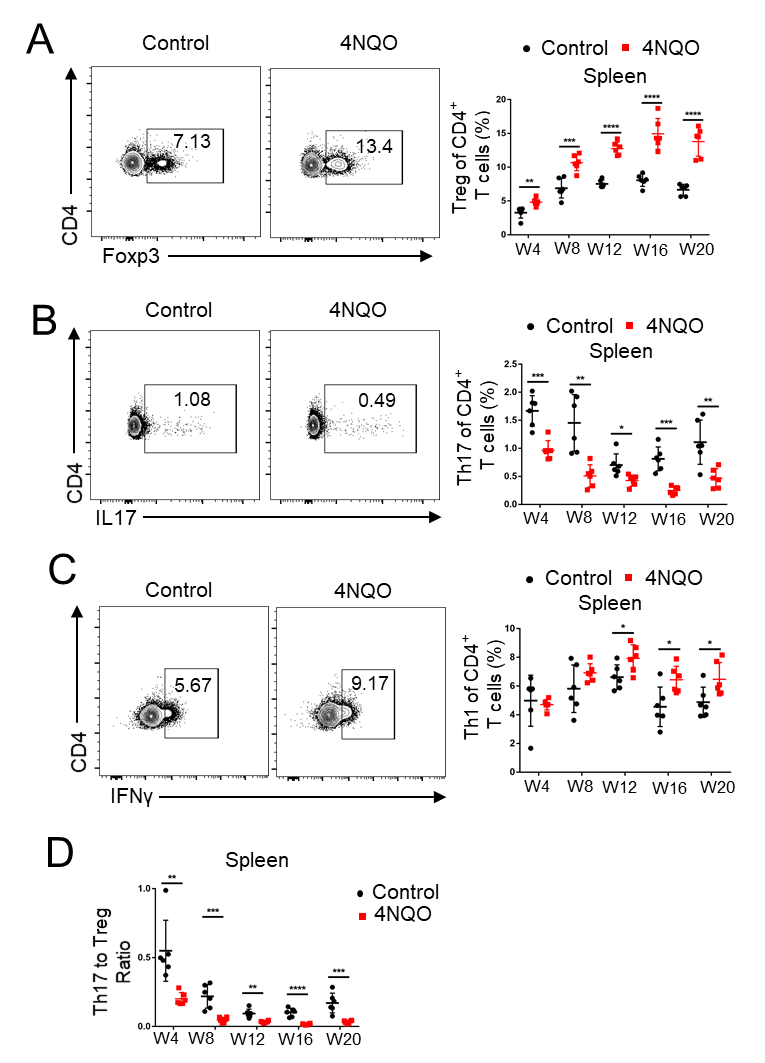
**

**Figure S5. The proportion of Treg and Th1 in spleen increased, while the proportion of Th17 decreased during the development of OSCC.** (A-C) Representative flow cytometric analysis and the percentages of Treg (A), Th17 (B) and Th1 (C) in spleen were shown. (D) Representative the ratio of Th17 to Treg were shown. W4, week 4; W8, week 8; W12, week 12; W16, week 16; W20, week 20. **p*<0.05, ***p*<0.01, ****p*<0.001, *****p*<0.0001.


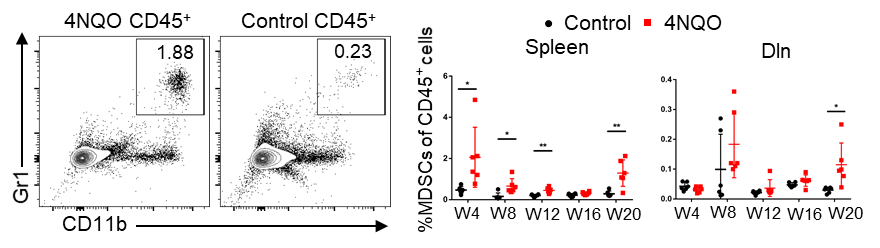


**Figure S6.** **Representative flow cytometric analysis and the percentages of MDSCs in spleen and Dln were shown.** W4, week 4; W8, week 8; W12, week 12; W16, week 16; W20, week 20. **p*<0.05, ***p*<0.01, ****p*<0.001, *****p*<0.0001.


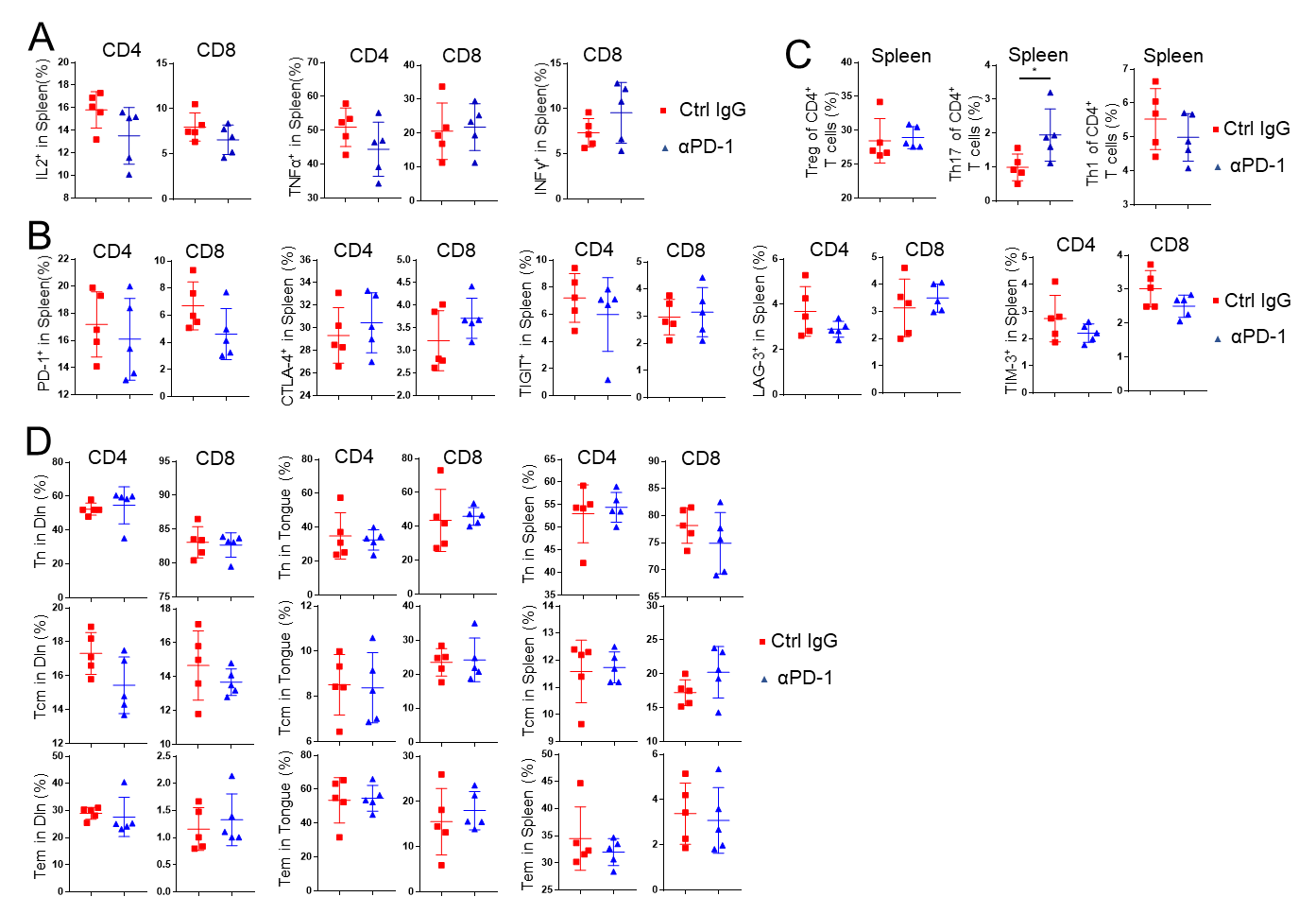


**Figure S7. Exhaustion features of T cells in spleen after αPD-1 treatment were shown.** (A) The percentages of T cells secreting IL2, TNFα and IFNγ in spleen of Ctrl IgG group and αPD-1 group. (B) Expression of PD-1, CTLA-4, TIGIT, LAG-3, and TIM-3 on T cells in spleen of Ctrl IgG group and αPD-1 group. (C) The percentages of Treg, Th17 and Th1 in spleen of Ctrl IgG group and αPD-1 group. (D) Statistical results of CD4^+^ and CD8^+^ T cell differentiation states in Dln, tongue and spleen. **p*<0.05.

**Table S1. Anti-mouse antibodies for flow cytometry**

| Flow cytometry antibodies | Source | Clone |
| --- | --- | --- |
| Anti-CD3-redfluor 710 | Tonbo Biosciences | 17A2 |
| Anti-CD4-Violet Fluor 450 | Tonbo Biosciences | Gk1.5 |
| Anti-CD8- efluor 506 | eBioscience | 53-6.7 |
| Anti-CD44-PercP-Cyanine 5.5 | eBioscience | IM7 |
| Anti-CD62L-PE-Cyanine7 | eBioscience | MEL-14 |
| Anti-PD-1-Brilliant Violet 710 | Biolegend | 29F.1A12 |
| Anti-TIGIT-PE | Tonbo Biosciences | 1G9 |
| Anti-LAG-3-APC | eBioscience | C9B7W |
| Anti-TIM-3-Super Bright 600 | eBioscience | 8B.2c12 |
| Anti-CTLA-4-PE-efluor 610 | eBioscience | Uclo-4B9 |
| Anti-CD4-PE-Cyanine7 | Tonbo Biosciences | RM4-5 |
| Anti-CD8-Brilliant Violet 702 | eBioscience | 53-6.7 |
| Anti-TNFα-PE | eBioscience | MP6-XT22 |
| Anti-IFNγ-PercP-Cyanine 5.5 | eBioscience | XMG1.2 |
| Anti-IL2-efluor 506 | eBioscience | JES6-5H4 |
| Anti-Foxp3-Brilliant Violet 421 | eBioscience | MF-14 |
| Anti-IL17-FITC | eBioscience | eBio17B7 |
| Anti-CD45-efluor 506 | eBioscience | 30-F11 |
| Anti-CD11b-FITC | Biolegend | M1/70 |
| Anti-Gr1-efluor 450 | eBioscience | 1A8-ly6G |
| Ghost Dye Red 780 (13-0865-T100) | Tonbo Biosciences |  |
